# Epstein–Barr Virus Uveitis Confirmed via Aqueous Humor Polymerase Chain Reaction and Metagenomics—A Case Report

**DOI:** 10.3390/medicina60010097

**Published:** 2024-01-04

**Authors:** Ning-Yi Hsia, Henry Bair, Chih-Ying Lin, Chun-Ju Lin, Chun-Ting Lai, Chieh-Min Chang, Jane-Ming Lin, Yi-Yu Tsai

**Affiliations:** 1 Department of Ophthalmology, China Medical University Hospital, China Medical University, Taichung 40402, Taiwan; deepwhite1111@hotmail.com (N.-Y.H.); hbair@stanford.edu (H.B.); u101001306@cmu.edu.tw (C.-Y.L.); withwind037@yahoo.com.tw (C.-T.L.); d4301@seed.net.tw (J.-M.L.); 2 Department of Optometry, Asia University, Taichung 40447, Taiwan; 3 Byers Eye Institute, School of Medicine, Stanford University, Stanford, CA 94303, USA; 4 School of Medicine, College of Medicine, China Medical University, Taichung 404327, Taiwan; 5 Precision Medical Center, China Medical University, Taichung 404327, Taiwan; fmingk@gmail.com

**Keywords:** Epstein–Barr virus, polymerase chain reaction, metagenomics, uveitis

## Abstract

This is a case report of Epstein–Barr virus (EBV) uveitis confirmed via aqueous humor polymerase chain reaction (PCR) and metagenomics. This 72-year-old male with a history of diabetes and herpes zoster complained of redness and blurred vision in his right eye for eight months. Mild conjunctival injection, anterior chamber cells, mutton-fat keratic precipitates, and vitreous haze were noted. Fluorescein angiography revealed dye leakage from retinal vessels without retinal ischemic changes. Only the serum anti-cytomegalovirus (CMV) IgG was positive while the aqueous humor PCR for VZV (Varicella-zoster virus), HSV (Herpes simplex viruses), CMV, and EBV was initially negative. Inflammation recurred and vitreous haze worsened after discontinuing nine-month topical ganciclovir and oral prednisolone. the aqueous humor PCR was repeated due to persistent low-grade inflammation. The EBV PCR turned out to be positive. Shotgun metagenomics revealed 1459 classified sequences (1.62%) and confirmed the EBV infection. Topical ganciclovir and methylprednisolone treatment was resumed. Conjunctival injection improved while pigmented keratic precipitates lessened. Elderly patients with diabetes or under immunosuppression may be susceptible to chronic uveitis associated with subsequent EBV infection. Repeated aqueous humor PCR and shotgun metagenomics are important tools in the diagnosis of this case of chronic indolent panuveitis.

## 1. Introduction

Epstein–Barr virus (EBV) is a ubiquitous DNA virus of the Herpesvirus genus. EBV DNA has previously been isolated from the ocular structures of normal eyes and from immunocompromised inflamed eyes [1,2]. The cellular immune response to EBV is mediated by both cytotoxic and helper T cells [3,4].

The infection of the eye by EBV can affect any segment of the eye, resulting in a range of associated oculoglandular syndromes, such as dry eye syndrome, dacryoadenitis, conjunctivitis, episcleritis, keratitis, choroiditis, retinitis, retinal vasculitis, papillitis, and uveitis [3,4,5,6].

Less than 1% of all microorganisms of the available environmental microbiota can be cultured with currently available techniques. Metagenomics is a new methodology for high-throughput DNA sequencing, able to provide taxonomic and functional profiles of microbial communities without the necessity of culturing microbes in the laboratory [7,8,9,10].

We report a case of aqueous humor polymerase chain reaction (PCR)-proven EBV chronic panuveitis not associated with systemic mononucleosis and confirmed via the shotgun metagenomics data.

## 2. Case Description

**Figure 1:**
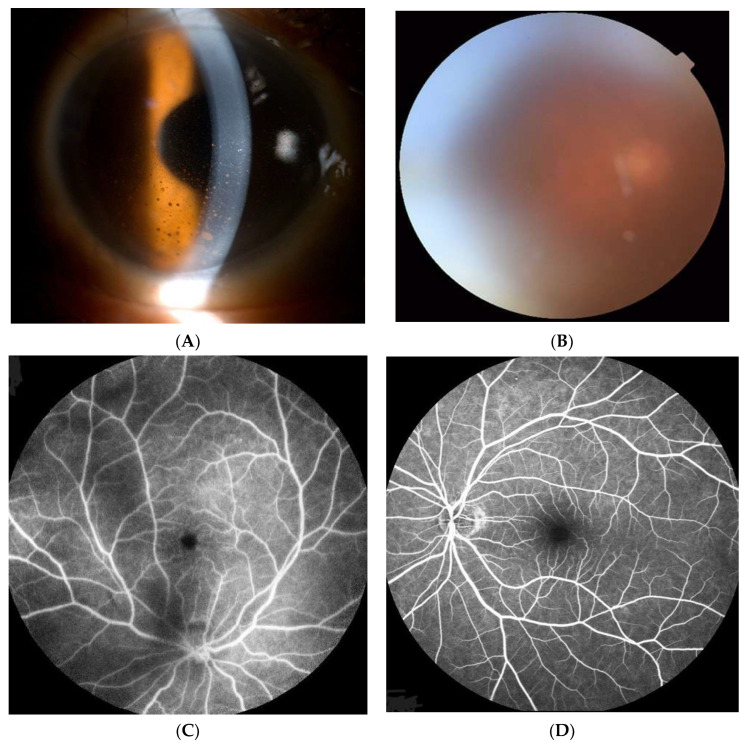
(**A**) Mild conjunctival injection, anterior chamber cells 3+, and mutton-fat keratic precipitates in the right eye (OD). (**B**) Vitreous haze 2+ OD. (**C**) Fluorescein angiography (FA) revealed dye leakage from the retinal vessels without retinal ischemic changes OD. (**D**) FA in the left eye was unremarkable.

A 72-year-old male was referred from a local eye clinic with a diagnosis of persistent uveitis for eight months. He had been treated prior to referral. He presented with complaints of redness and blurred vision in the right eye (OD). His past medical history included well-controlled diabetes with HbA1c about 6.6% for 5 years and herpes zoster along the back. His best-corrected visual acuity (BCVA) was 20/40 OD and 20/25 OS. Mild conjunctival injection, 3+ anterior chamber cells, mutton-fat keratic precipitates (Figure 1A), and 2+ vitreous haze were noted OD (Figure 1B). Fluorescein angiography (FA) revealed dye leakage from the retinal vessels without retinal ischemic changes OD (Figure 1C). FA in the left eye was unremarkable (Figure 1D).

The first impression was chronic granulomatous panuveitis OD. The systemic workup for the differential diagnosis of uveitis including blood tests and chest X-ray did not suggest any specific etiology such as sarcoidosis, intraocular tuberculosis, syphilis, or human T-cell leukemia virus type 1 (HTLV-1). Only the serum anti-cytomegalovirus (CMV) IgG was positive, so oral valganciclovir and intravitreal and topical ganciclovir were started for a presumed viral infection.

However, an anterior chamber tap fluid PCR at this time was negative for varicella zoster virus, herpes simplex virus I/II, CMV, and EBV. Therefore, oral prednisolone 20 mg was added. Oral valganciclovir had been given for 1 month. The vitreous haze gradually subsided to 0.5+, and oral prednisolone was tapered to 5 mg daily for better blood sugar control. Topical ganciclovir was also discontinued after nine months of use.

**Figure 2:**
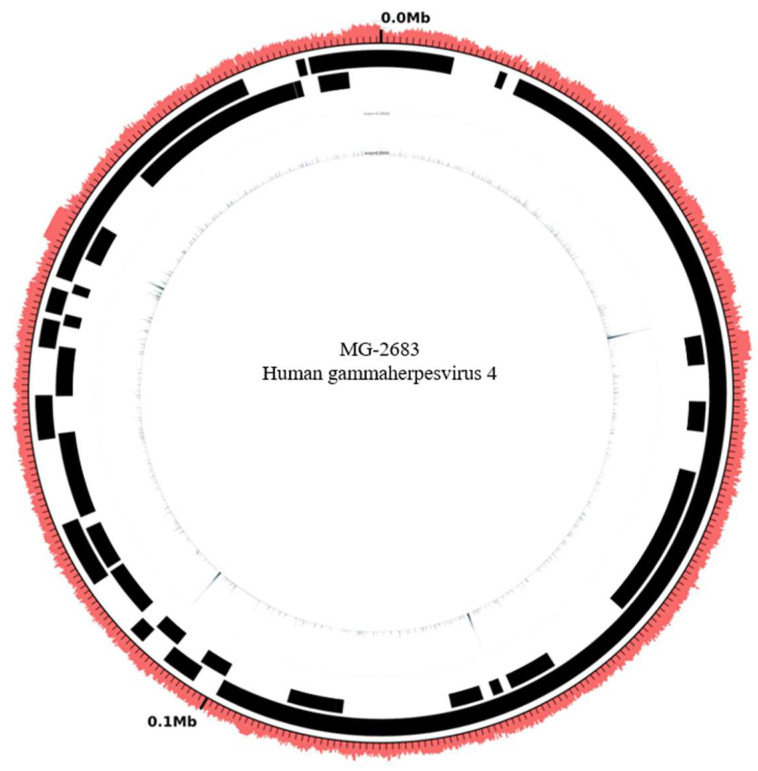
The outer circle represents the EBV reference genome sequence, while the inner circle displays the sequence and sequencing depth distribution of the patient’s sample. The uniform distribution of the sample’s sequence confirms the absence of false positive signals.

Unfortunately, the inflammation recurred a month after ganciclovir was discontinued, and vitreous haze worsened to 3+. Oral prednisolone was increased to 15 mg daily, and cyclosporin 200 mg was given simultaneously for three months with moderate response. The aqueous humor PCR was repeated due to persistent low-grade inflammation. Varicella zoster virus, herpes simplex virus I/II, CMV, and toxoplasmosis were still negative; however, the EBV PCR turned out to be positive. His shotgun metagenomics analysis revealed that 1459 sequences (1.62% of the total) were classified into specific microorganisms. After screening these sequences, it was confirmed that there was 169 EBV sequences in the sample are significant and correlate with the observed clinical symptoms and signs. We utilized sequences classified under the species (EBV) and mapped them to the reference genome through the Burrows–Wheeler alignment method. Visualization was then presented, with the uniformity of sequence distribution serving as a secondary verification to ensure the results were neither false positives nor interference signals (Figure 2).

**Figure 3:**
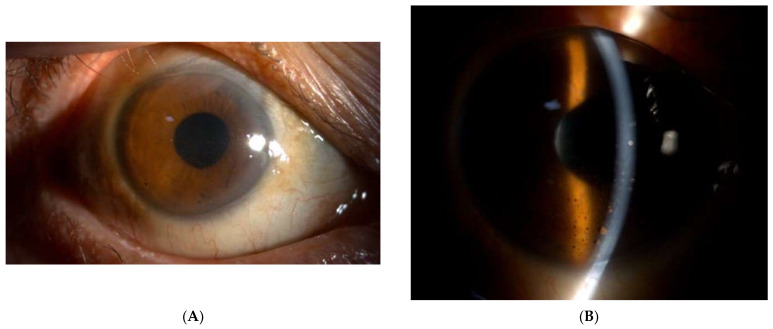
(**A**) Conjunctival injection improved. (**B**) Pigmented keratic precipitates was still noted.

Topical ganciclovir and methylprednisolone treatment were continued. Posterior subtenon 30 mg triamcinolone was given OD every eight weeks. Conjunctival injection improved (Figure 3A) and pigmented keratic precipitates lessened (Figure 3B). His BCVA remained 20/40 for one year after the last episode.

## 3. Discussion

A total of 95% of all people around the world have a EBV infection which occurs nasopharyngeally via either aerosol or direct contact; it tends to be asymptomatic in children but may be associated with the development of infectious mononucleosis in 50% or more of adolescents and young adults [3]. Several reports have identified EBV in patients with ocular inflammation [1,2,3,4,5,6]. However, given the widespread EBV exposure and its ability to remain latent in host cells, it has been difficult to clearly delineate its direct role as an ocular pathogen. In addition, EBV-associated uveitis has no specific characteristic features; therefore, we thought that the development of experimental tools and methodologies are important for EBV-associated uveitis diagnosis.

EBV-specific T cells can be suppressed via immunosuppressive therapy, allowing the spread of EBV to various organs, including the eye [2,4]. This patient was an elderly diabetic under immunosuppressive therapy (prednisolone and cyclosporin for about half a year). The late presence of EBV DNA in the aqueous humor may indicate an intraocular invasion of EBV-infected cells or a direct infection of EBV on the ocular tissues in this relatively immunocompromised patient.

Currently, only pre-specified pathogens can be detected using conventional culture-based techniques or PCR, but there are conditions to suggest that metagenomics could revolutionize the diagnosis of ocular diseases [7,8,9,10]. Clinical metagenomics is beginning to play an important role, not only in the identification of the normal flora of the human eye, but also in evaluating pathogens that may be important in ocular diseases.

For this experiment, the MGIEasy Serial Kit, in combination with the MGISP-100, was employed for library construction and circularization. The DNBSeq-50 sequencer was then used for sequencing purposes. The cell-free DNA (cfDNA) underwent processing into a library using MGIEasy Cell-Free DNA Library Prep Kit. As part of this processing using the MGIEasy Cell-Free DNA Library Prep Kit, the cfDNA underwent a series of modifications. This included an end repair reaction, adapter ligation to both ends through ligation reaction, and enrichment via amplification reactions. Following the construction of the library, the resulting material underwent circularization and transformation into a single strand DNB (DNA nanoball) using the MGIEasy Rapid Circularization Module. Subsequently, the prepared DNBs were subjected to sequencing on the DNBseq-50 platform, with each sample undergoing a 50-cycle single-end sequencing.

A read was removed if it contained more than 30% low quality bases (Q ≤ 2) or N bases. Kraken 2 (v2.1.1) was applied to analyze the metagenomics data [11]. The NCBI RefSeq database was used to annotate the sequence data against human, virus, archaea, bacterial, and fungal genomes (database version: July, 2020).

Our analysis process is mainly based on the NCBI RefSeq database for classification. When the similarity of a sequence is higher than 96% (the average sequencing read length used in this experiment is 48 bp), the sequence will be classified to the corresponding virus classification. The classification accuracy of this analysis method will be limited by the length of sequence data.

Metagenome sequencing was utilized to determine the presence of infections by uncommon pathogens. While cfDNA was used for the analysis to minimize interference from DNA of host cells, a significant quantity of host DNA sequences was still identified during the analysis. This necessitated a deeper sequencing depth to obtain an adequate amount of sequences for pathogen analysis. Additionally, the test made use of a previously established reference database with a sequence read length of only 50 nucleotides. Common errors and background noise were systematically filtered out during the analysis. However, due to the inherent constraints of the read length, there is potential for ambiguity in distinguishing pathogen sequences. It is noteworthy to mention that this issue was not encountered in our present study. Metagenome analysis using an ocular sample could possibly detect microbial genome and viral pathogens. Since *Propionibacterium* spp. are commensal bacteria found at the ocular surface, they are usually excluded from analysis.

This methodology has been validated in blood samples with concurrent viral load testing, showing consistent results with metagenomic positive pathogen samples. The occurrence of false negatives in blood is typically attributed to low viral loads. Currently, the sensitivity of metagenomics under limited sequencing depth is not as high as that of real-time PCR. The accuracy of this method for aqueous humor samples is not yet established due to insufficient sample numbers and the small volume of individual samples. A major concern with this approach is the risk of false positives. Developing strategies to filter out noise and avoid such false positives is crucial when identifying infections caused by unknown pathogens. The current methodologies employed in metagenomics face challenges in achieving quantification due to the absence of an added standard control. This makes accurate quantification more difficult.

EBV DNA was detected via shotgun metagenomics and confirmed via PCR in this case. Several antiviral drugs have been used to treat EBV infection, and the commonest antiviral treatments for ocular EBV infection are ganciclovir, valganciclovir, acyclovir, and valaciclovir. Several studies demonstrate the efficacy of oral valganciclovir, and the recommended regime is 450–900 mg twice daily for 6–12 weeks [12]. In our case, topical 2% ganciclovir was also given for maintenance therapy. To prevent the complications of uveitis, corticosteroids and/or immunosuppressive agents should be considered to decrease the inflammation of the eye.

Metagenome analysis requires specific system and instruments, and the PCR is decent for regular use. Metagenome analysis was considered for the patient who had persistent uveitis and poor response to current management, and/or negative results of the PCR for the common pathogen. Metagenome analysis could be used for further confirmation whether there is another uncommon pathogen, because it can detect a wider range of pathogens.

Elderly patients with diabetes or under immunosuppression may be susceptible to chronic uveitis associated with subsequent EBV infection. Repeated aqueous humor PCR and shotgun metagenomics are important tools in the diagnosis of this case of chronic indolent panuveitis. With the advances of clinical metagenomics, there could be a trend toward the use of mass sequencing-based diagnostics in the identification of ocular pathogens. The ability to identify live organisms will certainly require optimized collection and sample storage methods.

## Data Availability

There was no new data created, and the data is unavailable due to privacy.
